# Experiences of accessing maternity care in the UK: Perspectives from Somali migrant women in Leicester

**DOI:** 10.18332/ejm/143167

**Published:** 2021-12-13

**Authors:** Joan K. Konje, Justin C. Konje

**Affiliations:** 1 University of Warwick, Coventry, United Kingdom; 2 Obstetrics and Gynaecology, College of Life Sciences, University of Leicester, Leicester, United Kingdom

**Keywords:** migrant pregnant women, Somali women, genital mutilation, antenatal care, ethnic minority, access to maternity services

## Abstract

**INTRODUCTION:**

Migrant women born in Somalia often have poorer pregnancy outcomes. Access to care around pregnancy is vital to improve outcomes. The views and experiences of Somali migrant women accessing maternity care in the UK have not been extensively explored. This study therefore explores and describes these with regard to accessing maternity care in the UK, with the hope of gaining a better understanding of perceptions and factors that influence their access to care around pregnancy.

**METHODS:**

A qualitative approach was used to investigate the views and experiences of women born in Somalia who had migrated to Leicester. Data collected were transcribed and analyzed using a constant comparison method. The software package Nvivo 10 was used to organize themes, and verbatim quotes were used to support their interpretation.

**RESULTS:**

Main finings included: 1) positive attitudes of community midwives and availability lead to positive and meaningful experiences; 2) language difficulties and ineffective communication are barriers to effective access; 3) lack of cultural awareness and preconceived ideas by some hospital caregivers makes them unsupportive and insensitive; 4) need for continuity of care and the provision of resources are needed to build important trusting relationships with care providers; and 5) personal, community and religious factors impact access to care.

**CONCLUSIONS:**

The results highlight a number of important potential barriers to accessing care around pregnancy for migrant women born in Somalia and how these could be addressed to improve pregnancy outcomes.

## INTRODUCTION

It is estimated that there are about 115000 or more Somali born migrants living in the UK^1^, making it one of the largest Black African minority groups in the country. They are a diverse, vibrant, complex and heterogeneous community made up primarily of three groups; those born in the UK as descendants of merchant seamen of the 19th century, economic migrants and civil war refugee families, and asylum seekers (from Somalia and more recently migrants from mainland Europe)^2^. The recent inflow to the UK is drawn largely from the wider diaspora rather than those escaping Somalia itself and women and children make up the majority in this group^2^. Since 2000, these migrants (most of whom are now EU Nationals) have settled in different cities across the UK with an established Somali presence, for example in Leicester. Most of the women in this group neither speak their mother tongue nor English fluently, and consequently face significant challenges integrating into both the Somali and British communities in the UK^2^. While knowledge and understanding of the specific social and health needs of this community who are experiencing significant inequality in accessing education, employment, housing and health resulting in poor outcomes is increasing, it remains limited^2^.

Furthermore, the evidence from research highlights that pregnancy outcomes for Somali born women post migration in various European and other high-income (HIC) countries are poorer when compared with those of women in the receiving countries^3^. Reasons advanced for these differences include the physical, emotional and mental impact (including trauma) of migration and reluctance to access healthcare services during pregnancy (because of cultural insensitivity/awareness on the part of some of the providers) leading to compromised health and resulting complications^3,4^. An efficient and targeted maternity care for most of the women from this community is therefore vital.

Existing guidelines^5^ state that all pregnant women should have access to relevant information to enable them to make informed decisions based on their needs. These include access to free antenatal care, prenatal screening and information on food hygiene and supplements. Furthermore, they should receive the best support during antenatal care, childbirth, as well as post-delivery. The benefits of access (especially early access) to information and services about care during pregnancy and childbirth have been established elsewhere^6^. However, in the UK, women of black and minority ethnic communities including Black African migrants are generally more likely to access antenatal care late, receive fewer and or irregular antenatal check-ups or none^7^ and less likely to seek or be offered services such as anomaly scans or undertake prenatal screening^8,9^. There are suggestions that this lack of access to maternity care is a contributing factor to high maternal morbidity among these communities in the UK^9^ and indeed in other HIC countries^10^. Recent UK government guidelines recommend that all women have easy access to maternity care and continuity of care that is specifically designed to meet individual needs and those of the local (unit) community.

### Maternity care for Somali women

There is growing but limited evidence in the UK, Europe and other HIC countries such as the USA, Norway, Canada, New Zealand, and Australia, with large migrant populations on maternity care for women born in Somalia (hereafter referred to as Somali women). Few studies in the UK have identified several issues that impact on the maternity care for these women. Davies and Bath^11^ in their study on access to information during maternity care revealed that this was hindered by poor communication as well as inadequate knowledge about maternity care. A similar study of Somali refugees in London by Harper-Bulman and McCourt^12^ found that communication and language difficulties had negative implications on all aspects of their maternity care. These findings have also been reproduced in Norway^10^. In a more recent study of the perspective of Somali health workers in London, Straus et al.^13^ found that there was mismanagement of care for Somali women who had undergone female circumcision or female genital mutilation or cutting. Furthermore, midwives held stereotype and negative attitudes towards Somali women. Given their compromised health and possible complications, it is important that their views on access to care before and in pregnancy and during childbirth are explored.

The aim of this study was to explore and describe the views and experiences of Somali migrant women on accessing care before and during pregnancy in the UK and the factors that influence these, and on childbirth. A better understanding of these should not only be helpful in planning but also in delivering antenatal and intrapartum care to these women in the UK and in other high-income countries (HIC) with Somalis, and indeed large minority migrant groups.

## METHODS

This study used a combination of focus groups discussion and one-to-one semi-structured interviews in a qualitative approach underpinned by grounded theory^14^. This mixed qualitative method (one-to-one and focus groups interviews) was chosen to enable the generation of parallel accounts that complemented one another thus producing richer data and a more valid evaluation of findings. Focus groups were used to explore the women’s experiences of health services and their knowledge of health issues; it provided an opportunity for them to interact, ask each other questions, and re-evaluate and reconsider their understanding of specific issues and experiences. One-to-one semi-structured interviews provided a detailed and continuous narrative of their views on themes that emerged only briefly during the focus group discussions^15^.

A convenience and purposive sampling approach was used to locate, identify and recruit participants with similar characteristics that were of relevance to the study from Somali community centres^16^. Also, individuals were recruited to the focus groups through snowballing. The researcher contacted and liaised with managers of Somali community centers in Leicester and posted leaflets about the study. Prior to interviews and focus group discussions, participants completed consent forms and a brief demographic questionnaire that included personal details to establish length of residency in UK, number of children and age.

### Data collection

Two focus group discussions made up of five to six women were conducted in English and Somali with the help of an interpreter. This method is important because unlike traditional methods, it gives access to ‘hard to reach’ groups with inadequate literacy or language in the dominant culture an opportunity to participate in research^17^. A flexible moderator guide informed by the literature review was used to steer discussions enabling participants to express their views freely. The guide used included general and more specific questions relating to women’s experiences of accessing maternity care in the UK, the importance and take up of antenatal care and tests, their information needs around pregnancy and childbirth, their sources of support and information around pregnancy and childbirth, and suggestions to improve maternity care for them as a group. The guide was reviewed and updated after the first focus group discussion to reflect new information/issues that were emerging.

Five one-to-one semi-structured interviews were conducted using an interview guide at venues and times convenient to participants and these included their homes and places of work or study. These one-to-one interviews elicited private (individual) accounts of the everyday knowledge, experiences, thoughts and judgements around pregnancy and childbirth. These interviews provided a detailed and continuous narrative of each woman’s view on themes that emerged only briefly during the focus group discussions. All discussions and interviews were audiotaped.

### Data analysis

The audiotapes were transcribed and important themes and patterns defined. Collected data were then analyzed using a constant comparison method^16^. Data analysis began as soon as the first focus group discussion was completed. The data were transcribed and the transcript read over and over and as meaning was discerned, codes were applied to develop categories later used to develop one or more themes. The constant comparison method of grounded theory was used to compare data from different sources until data saturation was reached and main themes and sub-themes were developed^18^. At this stage, the data were imported into Nvivo 10 for organization into nodes (major themes). Finally, verbatim quotations were used (with language edits where appropriate) to illustrate the major themes identified by focus group and one-to-one interviews.

## RESULTS

A total of 16 participants aged 22–38 years who had all recently migrated to Leicester were studied. They had all recently accessed maternity care/services and were either currently pregnant or had had a baby in the UK within the last two years. Table 1 shows the demographic characteristics of the focus and interview groups. All the 6 women in the first focus group (FG1) spoke English fluently whereas the 5 in the second group (FG2) did not and therefore needed an interpreter. Some of the women had obtained EU citizenship in other countries (Netherlands, Denmark, Sweden) prior to coming to the UK while others were asylum seekers.

**Table 1:** Demographics of participants

*Participants*	*n*	*Mean age (years)*	*Mean number of children*	*Interview language*	*Years in UK*
Focus Group 2	5	30	3	Somali (interpreter)	Mean 6
Focus Group 1	6	28.5	3	English	Mean 9
Interview 1	1	38	4	English	10
Interview 2	1	24	2	English	9
Interview 3	1	30	5	English	11
Interview 4	1	27	2	English	6
Interview 5	1	34	4	English	5

Five themes on factors influencing access to maternity services emerged from the analysis of the data and these were: 1) positive attitude of community midwives and availability of community services; 2) language difficulties and ineffective communication; 3) lack of cultural awareness and preconceived ideas; 4) need for continuity of care and more resources; and 5) personal and community and religious influences, views and experiences of other Somali women.

### Positive attitude of community midwives and available community services.

Most women indicated that positive attitudes of the midwives in the community resulted in a positive maternity care experience. This was despite the stress from a shortage of midwives; they were still very friendly and caring:

*‘Our community midwife is very good and is always smiling. She is sometimes frustrated as she cannot visit us for more days.’* (Participant 5, FG1)

Some of the women developed trusting relationships with their midwives and consequently could speak freely with them and received advice. This experience was perceived as very useful and reassuring:

*‘I like the community midwives … they are very friendly … you almost forget that they are midwives. We see them as very caring and they let you speak to them confidentially; you feel like you have all the time to speak to them.’* (Interview 2)

*‘I got a lot of advice from the midwife and it was useful.’* (Interview 5)

Some of the women found the local Sure Start (a program targeting parents with children under the age of 4 years living in the most deprived communities in the UK) very useful. The women could get there easily and meet with others from their community and also talk freely about their health in general and their pregnancies in particular.

### Language difficulties and ineffective communication

Some women especially those having their first babies in the UK found the pregnancy booklets and leaflets given at the centers as sources of useful information. However, these were not perceived as very useful by the women who could neither read nor understand English. These contrasting views were highlighted in these quotes:

*‘At my first appointment with midwife I was given the pregnancy book and leaflets. As I could read, these provided information I needed and as such were very useful.’* (Interview 4)

*‘The welcome pregnancy package contains a lot of information about pregnancy and how to take care of yourself and your baby. However, if you cannot read English, that would be of no use to you.’* (Interview 2)

As mentioned above, some of the women mentioned that they could not speak English fluently making it difficult for them to communicate with health professionals especially in the hospitals. They were therefore gaps in their understanding even when the information was provided:

*‘How will I deal with a midwife in the hospital if we do not understand each other? What is the point of going there if she does not speak my language or understand me when I speak hers?’* (Participant 4, FG 1)

*‘Some women might not know what to do and because they do not speak the language and there are no information leaflets in Somali in our community where 70% of population here are Somali, they will therefore struggle to find them useful.’* (Participant 1, FG2)

Even without major language problems, poor communication can affect the care experiences of ethnic minority women. A subtheme that was very apparent was the importance of effective communication. Many women said healthcare staff did not adequately explain medications and investigations they were offered during pregnancy:

*‘If I have any questions, I will probably find somewhere else to go to than the GP. You are not told things in detail, they do not tell you anything relevant. I was offered DS (Down's Syndrome) testing and I accepted but there was no explanation, nothing. I was not even told clearly how the test was going to be done even though it was my first time.’* (Interview 1)

*‘When I first came and spoke little English they gave me a medicine when I was pregnant and did not explain what it was for.’* (Interview 3)

This lack of effective communication had a profound impact on the women during a stressful time of their lives. For example, at the hospital during delivery a woman described a situation where she was anxious about a procedure that no one explained and furthermore she felt completely ignored:

*‘About 10 different people came and looked at the baby monitor. I did not know what was happening, nobody was talking to me until I had to ask. They came and checked me, then they left; they did not inform me about anything or talked to me, nothing like that. Then they induced me. It was my first baby and they didn't explain why to me.’* (Participant 3, FG2)

In addition, the women were apprehensive of caesarean sections (CS) as these were not properly explained to them and some felt like they were being used for practice by some of the doctors:

*‘I got there in labor hoping for a normal delivery then at the last minute somebody told me, my baby was in distress and said sign here for a CS. I wanted to say no, hold your horse, what are you talking about? No one told me about the possibility of CS during delivery.’* (Participant 5, FG1)

Another subtheme was the lack of interpreters. It was seen as a barrier to accessing the maternity services. In the focus groups and one-to-one interviews, the women said they had to rely on someone from their community to interpret for them or cancel appointments if none was available:

*‘I needed someone to interpret for me as the midwife could not have the interpreter. I had to drag people from the community who then found out different things about me which was uncomfortable. Sometimes they had to cancel my appointments and I had to come back another day.’* (Participant 4, FG2)

*‘At the beginning (of delivery) I had to phone someone from my community to come to the hospital so I could get the needed help. I was uncomfortable with this and not happy; it made me a bit frustrated.’* (Participant 2, FG2)

### Cultural awareness and preconceived ideas

Many of the women commented on the lack of support and empathy from some of the health professionals especially hospital-based ones at a very vulnerable time of their lives and as a result avoided seeking help:

*‘After I had so many stitches, no one looked after me. I asked the midwife if she could help bring me tea and she said you have legs and can go and get it yourself.’* (Participant 2, FG 1)

*‘When you have that kind of attention (being ignored) you feel you are a burden and thus tend to avoid seeking help from the people you feel you are a burden on. You keep everything to yourself and want to get out of the place quickly.’* (Interview 04)

The women felt they were not being listened to during their interactions with staff, some of whom were ‘unfriendly’ and completely ignored the women. This was particularly so during labor, and that had an adverse effect on their experiences:

*‘They (staff) listen to the machine most of the time rather than to you.’* (Participant 4, FG 1)

*‘Sometimes you feel like you are being treated as an object; you are more or less given what they (staff) think you need and not what you feel you need; no one listens to you.’* (Interview 2)

Most of the women felt there was insufficient awareness or understanding of their culture by some health professionals to enable the staff to give appropriate care during childbirth. Most women felt that many midwives and health professionals were not well informed and trained on FGM/FGC, for example and on how to deal with women from their community who presented with it. This lack of awareness and resulting insensitivity led to very traumatic experiences and outcomes for some women:

*‘A lot of midwives do not know about our culture and when they see circumcision for the first time, (I think) they are shocked.’* (Participant 5, FG2)

*‘In our culture, women are sown up so it is difficult to deliver, so they have to be cut to get the baby out fast so the mum is not struggling. Here they did not cut and the baby was forced through causing severe damage to me. I requested a cut but no one listened.’* (Interview 1)

*‘I do not know why I had a caesarean section. It was not okay that they did not explain and I believe the doctors wanted to practice on me.’* (Interview 5)

Women felt that some health professionals had preconceived ideas and/or stereotype views about them and their culture and this sometimes led to abuse and insensitive and discriminatory comments:

*‘They have this perception that you do not require all these things because you are from this community or that Somali woman do not care about certain things like pain relief medicine.’* (Participant 1, FG2)

*‘Maybe because of our color, culture, religion or some other reason, they (staff) think they are better than us. Sometimes you will hear women being abused by comments like, you are coming back every day because you want to live on the benefit money. The worst thing one staff said was I will probably see you again next year … it makes you feel worthless.’* (Participant 2, FG2)

### Need for continuity of care and more resources

The women were frustrated at not being able to see the same health professional antenatally and at intrapartum, and stressed the need for continuity of care. Many, for example, found it very stressful meeting completely new individuals in labor (in the hospital) rather than the familiar and trusted community midwife they had confided in:

*‘People in my community have trust issues with anyone (strangers). They are very careful with whom they deal with. Besides giving birth in the hospital myself, I have also been there with people who needed my help and they questioned everything because they did not trust the staff.’* (Participant 4, FG1)

*‘In this country, when you become pregnant you see your community midwife and develop a trusting relationship with her, but when you are about to deliver you go to the hospital and meet a midwife for the first time who is new to you. At some stage of the labor where there are problems a doctor pops out from somewhere that you don't know.’* (Participant 5, FG1)

There was a feeling that maternity care in the community and in the hospital had been affected by a reduction in staff numbers and the number of available service days:

*‘Our community midwife used to work 5 days a week, but now this has been reduced to two, which is very stressful to her and us. She might thus be unable to give you as much time as you need because she is concern about the many people waiting in line to see her.’* (Participant 3, FG2)

*‘After delivery, I had to wait for two hours for stitches in the room where I had delivered my baby. I felt messy and nobody cared or came to clean me up and - blood was on the floor and everywhere.’* (Participant 3, FG1)

### Personal and community influences, views and experiences

Organizational factors as well as several personal and community factors were highlighted as barriers to the women accessing maternity care. These included their religious and traditional/community influences, fear of medical interventions and trust in the experiences of community women:

#### Religious and traditional/community influences

The influence of religion was reflected in the women’s views about accessing antenatal classes, tests and scans; referencing the perceived role of God "Allah" and religion in their healthcare seeking behavior:

*‘I was introduced to antenatal classes and stuff like that but I did not go. What is the point of going there; why do I need it anyway, (InshAllah) God will help me hence I know I will be fine. I don’t need all these breathing techniques.’* (Participant 2, FG1)

*‘Whether the baby is disabled, has Down Syndrome is going to be healthy or not, most Somali women refuse tests because they think whatever is inside, Allah created and they have to appreciate and accept it as a gift.’* (Participant 1 , FG 1)

Many women reflected on their personal as well traditional and community health beliefs around pregnancy, and how these impacted on their decision to access care:

*‘A lot of people belief that, the tests offered are done internally (vaginally) and they refuse them because they might miscarry. Anything that is optional tends to be rejected because they belief that the way the procedure is done maybe dangerous or risky.’* (Interview 2)

*‘In our community we tell each other things. My cousins are more educated, some have more kids than I do, so they know what they are talking about; they have more real-life experiences.’* (Participant 2, FG 2)

Some participants expressed an obvious lack of knowledge and access to information about the availability of antenatal care services such as antenatal classes in their area.

*‘I do not know what antenatal care classes are …’* (Interview 1)

*‘The women are just going to Sure Start every day and checking their blood pressure and baby's heart beat that's it, no one is informing them where to go for classes, what exercises to do during pregnancy …’* (Participant 3, FG2)

However, those who knew about antenatal classes thought these were irrelevant especially those having second or third babies. They thought they were only important for women having babies for the first time:

*‘Most women think it's a waste of time; they very rarely go because they will not learn anything new. When it is the first child the woman should go but when she has other kids she would not.’* (Interview 5)

*‘I do not go to them as they are optional. They are quite important if you are having you first child because they will give you an inside (to pregnancy and childbirth) and answer a lot of questions. I do not have time and it's my second child, so I have been through it once and kind of know what to expect.’* (Interview 2)

#### Fear of medical intervention or caesarean sections (CS)

All the women wanted everything done to enable them deliver normally. They were apprehensive about prenatal tests like Down Syndrome screening and interventions at delivery especially CS because of possible complications:

*‘If you have a CS, the place of the cut becomes weak and you cannot have more children. I think most of us believe in having more than one or two children. So that is the biggest phobia, I think. Because of the experience of a poor service, you will always believe that you did not get enough help and support and that is why they are going for the CS.’* (Participant 1, FG 2)

*‘Back home (in Somalia) there is no such thing as CS. A Somali woman with five or six children cannot afford to have a CS with complications and stay in bed for 6 months after.’* (Interview 4)

#### Trust in the experiences of and advice from community members

The women trusted and relied on advice and support from their mothers and other women in their community during pregnancy than from the health professionals. Women in the community could be trusted easily more so because of their extensive experiences:

*‘My mother has been through it before so I ask her questions. I do not bother going to the doctors, I put it off. I do not see the midwives often. Any concerns or worries that I might have I might call on someone experienced who has had 4 children and ask. There are however, a lot of myths and you have to separate them from facts.’* (Interview 2)

### Suggestions to improve awareness and access to services

The women suggested ways to increase awareness and access as well as improve existing services around pregnancy. The suggestions were focused on communication, cultural awareness and service improvement:

#### Appropriate language and effective communication

It was suggested that the language and method used to communicate with them needed to be appropriate, and the information delivered effectively using professional interpreters (i.e. independent – not relatives or from the community) and be available for those who were not fluent in English:

*‘I will benefit more and get better services if they (staff) had a person who spoke my language.’* (Participant 4, FG1)

*‘I think they (staff) should get some interpreters to translate … it will be better for establishing good relationships and communicating well.’* (Interview 4)

They emphasized that health professionals should communicate more clearly and effectively with them and that the tools of communication should be appropriate:

*‘Even if the leaflet is in my language, it should understandable. It should contain target words, not too long and with easy-to-understand content.’* (Participant 5, FG1)

#### Use of community groups with peer support

Participating women felt that group meetings or discussions, that were women-only and led by or involved someone familiar with or from the community would be very useful and an effective communication tool:

*‘Group meetings are better … only women meetings like this one. Both young and elderly should mix.’* (Participant 2, FG2)

*‘The best way to inform women about services is to get someone from the Somali community that we are familiar with as we tend to believe someone if they are older. (They) can come along with the midwife/doctor and act like the middle-man between them and the community. Because if it is somebody outside the community, I do not think they will be taken seriously.’* (Interview 2)

#### Improve cultural-sensitivity staff training

In terms of improving cultural sensitivity in the care provided, the women suggested that staff should be trained on issues related to ethnic and cultural diversity:

*‘Staff should be trained in equality and diversity as they do not understand other people’s culture, background and how they think. They (staff) need to be trained.’* (Participant 5 , FG 1)

*‘They (staff) need to know more about our culture and relate to us as human beings and not as something else … the staff have to be educated properly and have people skills and not criticize us and put us down for having too many children.’* (Participant 1, FG2)

#### Educating health professionals around FGM/FGC

It is important that health professionals are educated on FGM/FGC and be opened to help affected women. Most in the study had undergone FGM/FGC and were comfortable talking about it. They felt that it was a good idea to work with health professionals to manage FGM/FGC before pregnancy and that it was their responsibility to seek help:

*‘If you already had period problems and felt pain as a teenager, you should seek help for this so that you are open-up before marriage if that is what you need.’* (Participant 1, FG1)

*‘It is good to have contact with the doctor before you even get pregnant because of the consequences that will come after.’* (Participant 3, FG 1)

Some women offered suggestions about how to build trusting relationships between staff and the women, which was perceived as very important to them and would ensure continuity of care:

*‘You should have one or two people that will deal with you basically throughout pregnancy and delivery.’* (Participant 3, FG2)

## DISCUSSION

This study identified five main themes that had an impact on understanding of and access to maternity care services by Somali women in Leicester. These included: 1) impact of available community service and attitude of community midwives; 2) language difficulties and ineffective communication; 3) cultural awareness especially of FGM/FGC by hospital staff; 4) trust and continuity of care; and 5) personal, community and religious beliefs and views.

Care from the midwives in the community and Sure Start centers was rated highly, and positively impacted on experience of accessing maternity care. The midwives were described as friendly, caring and trustworthy people to whom the women could talk freely and confidentially about their health and the pregnancy. These findings are similar to those from similar studies in Norway and the UK that identified the link between the trust gained by women with care providers and their access to healthcare services^10^.

Although the pregnancy booklets the women received from the midwives at their first antenatal visits in the community centers were acknowledged to contain very useful information on pregnancy and childbirth, many felt language barriers significantly impacted on their benefits to the women. This was more for those not fluent in reading or speaking English. The women suggested that translating these into their language or using information in them at discussion fora/groups could better disseminate it. Other studies^10,12^ have also highlighted the linguistic concerns of recently arrived non-European language speaking women and the lack of adequate provision of language support services. This affected the ability of some of the women in this study to access maternity services as well as making informed choices and decisions about their care. Similar findings have also been reported elsewhere in studies with or including Somali women^13,19^. Although it is well established that effective communication, particularly between healthcare professionals and patients, is a fundamental prerequisite for quality in maternity care, its importance seems not to always be emphasized in most settings^20^.

Compounding the language difficulties in our study was inadequate explanation to the women of why things were being done by healthcare providers. This led to a lack of understanding of procedures such as CS or tests like Down syndrome screening, all of which negatively impacted on the care received. Dormandy et al.^20^ in studies of migrants and access to antenatal care that included Somali women also found lack of understanding about procedures as a barrier to accessing care. A study by the Tower Hamlet Primary Care Trust in London made several recommendations on how to improve access including the need for proper explanations of test procedures and screening, and for all information in the ‘red’ book (pregnancy booklet) to be explained to women. The women we studied did not only make similar suggestions but also highlighted the need for available, simple, step-by-step and easily understood information, preferably in their own language. Evidence clearly demonstrates that women have better physical and emotional outcomes during labor if they understood the care, they were receiving^21^.

Lack of independent professional interpreters was an important factor in the cancellation of clinic appointments with midwives in the community in this group. The use of professionally accredited interpreters has been shown to improve both access to care and clinical outcomes for those with limited English proficiency^21^. In the UK, official maternity service policies require all providers and primary healthcare centers to offer professional interpreting services to those with limited or no English^5^. It has been shown that lack or shortage of independent interpreters might diminish the desire of women to access antenatal care early or regularly^11^. Reliance solely on interpretation as the method of overcoming communication barriers fails to acknowledge that many migrant women, including Somali women, are reluctant to use interpreters for fear of misinterpretation and breach of confidentiality, if these are from their communities^10^. The women we interviewed said they were not happy that friends and family members were used as interpreters because of these concerns. While advocating for independent interpreters to be made continuously available, our findings suggest a need for these to be culturally trained and also be aware of the specific needs of women.

A key factor to a better pregnancy outcome is continuity of care. In this study, the women had very positive views towards the community midwives with whom they had established a trusting, understanding and caring relationship. This contrasted with the insensitivity and stereotype treatment shown by some of the hospital staff they met for the first time in labor. It has been shown that women from ethnic minority communities are less likely to have access to continuity of care, even though this may be more important for them^22^. The UK government guidelines state that throughout pregnancy and childbirth, midwives are responsible for ensuring continuity of care in order to increase women’s satisfaction, improve communication and enhance overall sense of control and the ability to make informed choices^23^. In studies involving Somali women, lack of continuity of care led to severe communication difficulties and made it harder for the women to develop vital relationships and rapport with health professionals that required familiarity and trust^11-13^. The women in this study were frustrated by lack of continuity of care and would have preferred the midwife they knew and trusted in the community to be present at childbirth in the hospital. Meeting staff for the first time in the hospital and especially in labor did not allow them enough time to establish trusting relationships that could positively impact on the care received. While the women advocated for a designated community midwife to be part of their birthing team, it is important to acknowledge the implications of this on staffing levels and numbers^23^. Indeed, lack of enough midwives in the community meant appointments were overbooked, some cancelled and available clinics reduced from 5 to 2 per week.

In this study, women viewed the attitudes of some staff towards them as generally poor. Many felt frustrated by the rudeness, lack of understanding and empathy (towards them) from some hospital staff and this negatively affected them. Some of the experiences left the women traumatized and feeling worthless. That their views and opinions were not listened to, that they were not involved in decision making about their birth and felt completely ignored, were findings previously reported^24^. Cultural insensitivity, labelling, prejudice and stereotype comments and discrimination by care providers negatively affect care received, as was the case here and from other studies exploring access to maternity care and child birth experiences of migrant women, including Somalis^10^. The women felt devalued and alienated by some staff who stereotyped them and/or treated them without sensitivity^25^.

Poor knowledge, lack of cultural sensitivity and inconsistency in care by some staff around FGM/FGC were concerns of the women. Some midwives lacked basic knowledge and awareness on how to deal with FGM/ FGC at delivery. Consequently, they provided insensitive care making labor traumatic to the women. Furthermore, complications like perineal lacerations were blamed on this. These findings are in line with those from other studies of similar women^26-28^. In addition, our women identified as an issue inconsistency in their care of women with FGM/FGC across the country. Defibulation, for example, was not routinely offered in Leicester, but our cohort were aware that it was offered in London. The women suggested that hospital staff, especially midwives, should be offered indepth training on cultural diversity in general and how to deal sensitively with issues around FGM/FGC in particular.

Religious beliefs have been shown to play an important role in the health seeking behaviors of Somali and Muslim women^29^. This was acknowledged in this study where the women expressed a strong belief in God’s role in their lives. This influenced their decision to seek information about or undertake certain procedures, especially those related to identifying abnormality (as termination of pregnancy is not allowed in their religion which holds that unborn children should be accepted as a gift from God without question). Most, therefore, did not have antenatal DS screening. Recognizing these beliefs and counselling appropriately, for example that screening does not equate to termination of pregnancy, would be more acceptable and thus may increase acceptance.

The finding that the women held very negative views and attitudes towards caesarean delivery, such as fear or apprehension of the procedure, general dissatisfaction and skepticism, is consistent with published studies^10,11,13^. They were apprehensive of CS because of the perceived greater risk of complications that could stop them from having more children and/or recover quickly to care for their babies. While this is not unique to this population, it highlights the importance of clear communication and discussion of the benefits, risks and indications for procedures or interventions like CS, not only to Somali women but to all pregnant women and their partners, antenatally and intrapartum, to help them make informed choices^30^. The mistrust among the women of the medical motives for CS was based on the common belief that inexperienced doctors in hospital use women like them for practice as previously reported^31^ and was partly a consequence of either being misinformed or inadequately informed.

Interestingly, certain common traditional beliefs and views, as well as experiences of others within their community, influenced the women’s decision to access care. There was more reliance on community support and the experience and expertise of trusted women in the community than on healthcare professionals. Some women ignored or did not engage regularly/properly with maternity services because of lack of trust in healthcare professionals as a result of past negative personal experiences^10^. Conversely, the women trusted the advice of experienced community (older relatives) women whom they knew very well. Unsurprisingly, they suggested that maternity information and advice would better be disseminated through group discussions led by or with the involvement of familiar and respected members of the community with whom they could relate, acting as ‘middlemen’ as previously shown^10^.

### Implications for practice

If access to maternity services by migrant women such as those born in Somalia is to be improved, the findings from this and other studies show that there must be effective communication between the women and healthcare services. This can only be achieved if language difficulties are overcome. This would be possible through the use of independent professional specific interpreters who are culturally trained and are available antenatally and intrapartum. Where possible documents such as pregnancy booklets, patient information leaflets and other educational materials should be translated into the language of minority groups, as suggested by the women in this study. This must, however, be rationalized based on resources and the population being targeted as not every minority group could be offered this.

The role of community discussion groups led by or supported by peer educators should be explored as an approach to raise awareness of and disseminate information about maternity care within this particular community. The community and its members to whom the women relate better, and trust more, are an important resource in educating and empowering women about their health in general and in pregnancy.

Cultural competence training programs, particularly on diversity, for all healthcare professionals should not only be improved but made mandatory and furthermore effectively and regularly monitored. Such training programs should focus on finding ways to build trusting relationships, particularly between hospital-based staff (such as those in delivery units) and the women, to improve their delivery experiences.

Research should be undertaken into possible involvement or role of African migrant men in the care of their pregnant wives. Although this study did not focus on this issue, it was raised as a concern by some women.

### Limitations

Although the number of volunteers in this study could be considered small and therefore a weakness, most such studies have samples of 6 to 10. Furthermore, the 16 women that had had several pregnancies and the fact that many had all delivered in Europe, makes their observations very powerful in the context of the UK and other countries receiving migrants from Europe. Being a UK-based study could also be considered a limitation, but we feel that the experiences of migrants in high-income countries, worldwide, would be the same as reported by Utne et al.^10^ from Norway.

Purposeful and convenience sampling was used to recruit the participants which meant that they did not represent the whole community in the UK and this might have introduced selection bias^32^. However, although the sample was selected purposively, they were used purposefully to analyze the data collected, in order to illuminate subtle but potentially important differences^33^. The use of a qualitative approach underpinned by grounded theory enabled us to gain a different and deeper perspective about access to maternity care among Somali women, many of whom had migrated from other European countries. Indeed, some of the factors that influenced access to maternity care, highlighted by the women in this study, were similar to those of Somalis in Norway^17^. The use of interpreters could have potentially introduced an element of bias in the deciphering of the data, however as most of the translated data were similar to those obtained from women who spoke English fluently, this is unlikely to have been significant. In this context, it is recognized that interpretation of attitudes of staff by the women may reflect perceived stereotypes in the communities rather than reality.

## CONCLUSIONS

This study identified 5 key broad factors that influenced access to care before and around pregnancy by Somali migrant women in the UK. These were communication difficulties, cultural awareness or lack of it, influence of communities (discussions and opinions of others), and need for continuity of care and support from community midwives. While the attitude of community midwives had a very positive impact, poor communication and lack of cultural awareness as well as insensitivity by some hospital staff significantly affected experiences to the extent that some women were traumatized and felt devalued. Increasing community midwifery services, providing professional interpreters trained in cultural and racial diversity and including community leaders in discussions and communications will go a long way to addressing some of these barriers.

## Data Availability

The data supporting this research cannot be made available for privacy reasons.
